# Meta-analysis of Asymptomatic Monkeypox Virus Prevalence in Nonendemic Regions, 2022–2024

**DOI:** 10.3201/eid3210.260305

**Published:** 2026-10

**Authors:** Kelsey J. MacCuish, Robert Snyder, Kelly A. Johnson, Edwin Chojolan, Awa Keinde, Kathleen Jacobson, Kayla Saadeh

**Affiliations:** California Department of Public Health Office of Sexually Transmitted Infections and Hepatitis C Virus, Richmond, California, USA (K.J. MacCuish, R. Snyder, K.A. Johnson, E. Chojolan, A. Keinde, K. Jacobson, K. Saadeh); University of California, San Francisco School of Medicine, San Francisco, California, USA (K.A. Johnson)

**Keywords:** mpox, monkeypox virus, sexually transmitted infections, asymptomatic, infection, detection, meta-analysis, viruses, United States

## Abstract

In this meta-analysis, we performed a literature search for articles relating to asymptomatic monkeypox virus (MPXV) detections published worldwide during May 12, 2022–September 13, 2024. The primary outcome was to determine the pooled prevalence of asymptomatic MPXV detections. We conducted a random-effects analysis that yielded a final pooled prevalence of 1.0% (95% prediction interval 0.1%–11.2%). Results did not substantially differ by geographic region or study design. Although those findings are reassuring, we did not evaluate infectivity or transmission potential among asymptomatic persons. MPXV continues to circulate globally; the viral dynamics that enable its persistence remain poorly understood and merit further study.

Clade IIb of human monkeypox virus (MPXV), which causes mpox, an infectious disease in the same family as smallpox, began spreading globally in 2022 and prompted international public health concern (*1*). MPXV can be transmitted through close or intimate contact between humans, through infected animals, or through fomites (*1*). In the United States, most MPXV infections are spread person-to-person through sexual contact, primarily among gay, bisexual, and other men who have sex with men (GBMSM) (*1*). The emergence of the disease in the United States and Europe, where clinical and epidemiologic resources are more abundant than sub-Saharan Africa, has improved our understanding of the clinical manifestations and epidemiologic characteristics of MPXV. It is now understood that MPXV can be transmitted in 1–4 days before a person has onset of symptoms of infection (*1*). However, whether persons who remain asymptomatic throughout the course of infection can transmit MPXV to others is much less clear. This uncertainty arises partly because persons need to have lesions to be clinically tested for MPXV in the United States. The prevalence of asymptomatic MPXV infection in the ongoing global outbreak also is unknown and could have important implications for public health efforts.

A previous systematic review and meta-analysis of the prevalence of asymptomatic MPXV detections estimated a pooled prevalence of 10.2% (95% CI 2.5%–17.9%) but only included reports available through September 2022 (*2*). Because most studies in that meta-analysis preceded the 2022 global MPXV outbreak and the analysis also included a report from an outbreak that began in a primate sanctuary in Cameroon, the results may not be generalizable to the ongoing outbreak that primarily affects GBMSM (*2*).

The absence of asymptomatic MPXV detections in study populations has been noted in numerous reports (*3*–*8*). For example, a retrospective study noted that only 0.2% of patients tested through PCR were asymptomatic. However, that study was conducted in the summer and fall of 2022, the peak of the global outbreak, when transmission was more widespread in the GBMSM community (*9*). In contrast, 2 studies that assessed the prevalence of asymptomatic MPXV detections among GBMSM in France and Belgium retrospectively screened anorectal, urine, and oropharyngeal samples and found higher prevalences of asymptomatic detections (5.5% in France and 1.3% in Belgium), although a notable methodologic limitation of those studies is that detection of viral genetic material does not necessarily mean that a person is infectious, whereas a negative test result indicates that infectiousness is highly unlikely (*10*,*11*). Because overlooking asymptomatic viral infections, if highly prevalent, could hinder disease control and prevention, we assessed the prevalence of asymptomatic MPXV detections worldwide through a meta-analysis of peer-reviewed articles published during May 12, 2022–September 13, 2024.

## Methods

### Search Strategy and Selection Criteria

We searched PubMed for relevant articles by using the search terms (asymptomatic OR non-lesion OR no lesion* OR without lesion* OR without cutaneous lesion* OR undetected OR unrecognized OR proctitis OR undiagnosed OR no symptom*) AND (monkeypox OR mpox OR MPXV OR MPX OR monkey pox OR monkeypox virus OR MPV). Because PubMed contains relevant biomedical literature related to MPXV and because of resource constraints and the substantial overlap between PubMed, Web of Science, and Embase, we opted for using PubMed as our primary database. We found additional relevant articles through citations from screened studies. We limited results to publication dates during May 12, 2022–September 13, 2024, to yield results relevant to the ongoing outbreak.

### Data Extraction and Management

The primary outcome was determination of the pooled prevalence of asymptomatic MPXV detections among persons tested for MPXV. We used the Preferred Reporting Standard of Systematic Reviews and Meta-Analysis (PRISMA) checklist to evaluate the searched articles (Figure 1). We did not prospectively register the review protocol. The first author began by screening and removing studies whose titles did not indicate asymptomatic MPXV detections; she also briefly reviewed abstracts of excluded articles. She then screened the abstracts for inclusion and exclusion criteria, after which 2 other authors independently reviewed the remaining full-text articles for inclusion. If discordance about whether to include an article occurred, we consulted a third co-author to determine whether the article met eligibility criteria.

**Figure 1:**
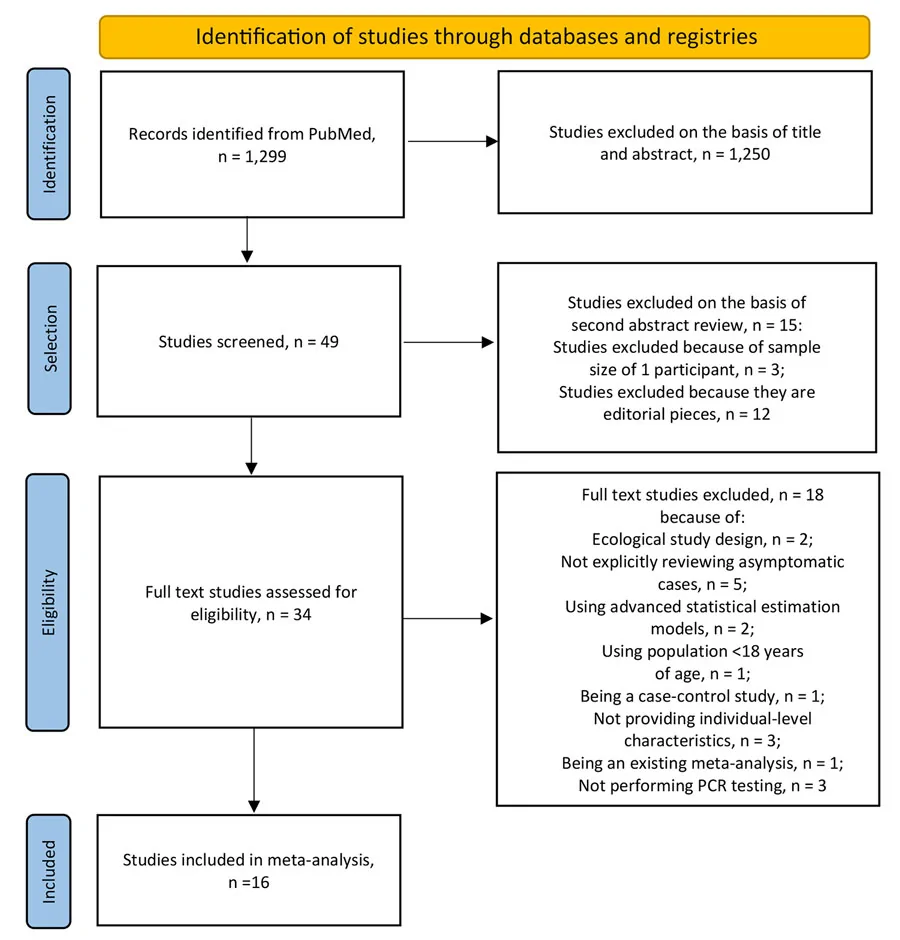
Preferred Reporting Standard of Systematic Reviews and Meta-Analysis flowchart for studies published during May 12, 2022–September 13, 2024, that were included in a meta-analysis of asymptomatic monkeypox virus detections in nonendemic regions.

### Inclusion and Exclusion Criteria

Studies about asymptomatic MPXV detections published during May 12, 2022–September 13, 2024, were eligible for inclusion. We followed the PICO (population, intervention, comparison, outcome) framework to synthesize our research question. We searched for articles that assessed individual adults (population) who tested positive for MPXV by PCR. PCR testing for asymptomatic persons enrolled in those studies was conducted by using pharyngeal, anorectal, urethral swabs, urine, or blood. We compared those asymptomatic persons who tested positive for MPXV to those who tested negative for MPXV (comparison).

We used the following inclusion and exclusion criteria and completed the JBI (formerly Joanna Briggs Institute) critical appraisal checklist for prevalence studies to determine whether studies should be included in the meta-analysis. The study must have been conducted during May 12, 2022–September 13, 2024; taken place outside of sub-Saharan Africa (in nonendemic regions for MPXV); been primary research (not a review of already published studies); must have had a population of >1 person; been conducted on the individual patient level (e.g., not an ecologic study or a study that does not provide counts of individual characteristics); researched people >18 years of age (e.g., any sex, sexual orientation, or race/ethnicity); researched transmission between humans only; not selectively sampled on MPXV infection (e.g., the study did not only include persons with MPXV); performed PCR testing to determine the presence of MPXV infection; and included persons who displayed no physical symptoms of MPXV as determined by explicitly stating that persons were asymptomatic, undetected, clinically inapparent, did not experience lesions or rashes, or a combination of these descriptions. Any mention of symptomology excluded a person from being asymptomatic or (if it did not otherwise include asymptomatic MPXV) the entire article from inclusion.

We initially considered vaccination status as inclusion criteria but ultimately did not include the criteria because very few studies accounted for vaccination status. We then excerpted the following from each included article: author (or authors), study design (cross-sectional, prospective cohort, retrospective cohort, mixed cohort), publication date, study recruitment dates, country where the study participants were recruited, number of asymptomatic MPXV patients in the study population, and total number of persons who were tested for MPXV in the study population.

### Data Analysis

We calculated the prevalence of asymptomatic MPXV detections as the cumulative number of asymptomatic MPXV detections divided by the total number of study participants tested for MPXV. We first constructed a funnel plot of the included research studies and statistically tested for biases and then estimated the pooled effect by using random effects estimates and their associated 95% prediction intervals (PIs) (*12*). We built logistic regression models to calculate random effects estimates, which assume that included studies estimated different interventional effects that follow an approximately normal distribution, and implemented a continuity correction of 0.5 for studies without any events. Our models used a logit transformation before pooling with the restricted maximum-likelihood estimator for between-study variance. To estimate the 95% PIs, we used the τ statistic that quantifies the variance between effect sizes, which, in this case, was the observed proportion of asymptomatic MPXV detections across studies. We used the 95% PIs to predict the underlying effect that would be seen in a hypothetical study similar to the studies included in the meta-analysis.

We conducted sensitivity analyses by using meta-regression to determine whether the prevalence of asymptomatic MPXV detections differed by geographic region or study design. We defined regions by continent and included Asia, Europe, and North America. Study designs included cross-sectional studies, mixed cohorts, prospective cohorts, and retrospective cohorts. If a study could not be grouped into a mutually exclusive category for either of those potential effect modifiers, we excluded it from that specific sensitivity analysis.

Finally, we quantitatively assessed between-study heterogeneity using the ι^2^ test, which measures the percentage of variability between studies that is not attributable to chance (*13*). We classified heterogeneity as low (<25%), moderate (25%–50%), and high (>50%). We conducted all analyses by using the meta and metafor packages from R version 4.4.1 (The R Project for Statistical Computing, https://www.r-project.org) (*14*,*15*).

## Results

Our search criteria yielded 1,299 articles, of which we excluded 1,250 (96.2%) based on the titles, which did not include information about asymptomatic MPXV or MPXV transmission (Figure 1). We also reviewed the abstracts of those excluded articles to ensure they did not include information about asymptomatic MPXV detections. We screened the abstracts and introductions of the 49 remaining articles and excluded 15 (31%) that did not meet inclusion criteria; 3 (6%) of the excluded articles described individual MPXV patients, and 12 (24%) were not primary studies (i.e., they were descriptive reviews, editorials, or commentaries). We then reviewed the remaining 34 full-text articles and excluded 18 (53%) because they either were ecologic studies (n = 2), did not explicitly study asymptomatic MPXV detections (n = 5), used modeling to project the number of MPXV detections (n = 2), studied a population <18 years of age (n = 1), were a case–control study that selectively sampled on MPXV case status (n = 1), did not provide individual-level characteristics (n = 3), were an already existing meta-analysis that included studies of primate sanctuary workers with MPXV (n = 1), or did not perform PCR testing to determine MPXV infection (n = 3).

A total of 16 articles met all inclusion criteria. One author compiled the JBI checklist for all included studies and a second author validated the checklist (Appendix Table). Of the included studies, 8 were prospective cohort studies, 2 were cross-sectional studies, 5 were retrospective cohort studies, and 1 was a mixed cohort study (i.e., containing prospective and retrospective cohorts) (Table 1). Three studies were from the United States, 3 from Belgium, 2 from Germany, and 1 each from Austria, Denmark, England, France, Italy, Japan, Spain, and Switzerland. Of the 16 included studies, 14 reported the number of male participants (Table 1). The study populations ranged in size from 25 in the cross-sectional study by Brosius et al. (*17*) to 6,600 in the cross-sectional study conducted by Koppe et al. (*21*).

**Table 1:** Summary of studies included in meta-analysis of prevalence of asymptomatic MPXV detections in nonendemic regions*

Authors and year (reference)	Study design	Country	Sampling dates	Recruitment sites	Specimen type	No. (%) men	Asymptomatic MPXV detections	Sample size
Agustí et al. 2023 (*16*)	Cross-sectional	Spain	2022 Aug–2022 Oct	Community center	Pharyngeal swabs, anal swabs	89 (78.8)	7	113
De Baetselier et al. 2022 (*11*)	Retrospective cohort	Belgium	2022 Jan–2022 May	Sexual health clinic	Pharyngeal swabs, anal swabs	224 (100)	3	224
Brosius et al. 2023 (*17*)	Prospective cohort	Belgium	2022 Jun–2022 Jul	Sexual health clinic	Pharyngeal swabs, anal swabs, blood	24 (96.0)	2	25
Contag et al. 2023 (*18*)	Retrospective cohort	USA	2022 Apr–2022 Sep	Public hospital and clinic system	Pharyngeal swabs, anal swabs, urine		4	1,645
Ferre et al. 2022 (*10*)	Retrospective cohort	France	2022 Jun–2022 Jul	Sexual health clinic	Anal swabs, urine	200 (100)	11	200
Golden et al. 2024 (*19*)	Prospective cohort	USA	2022 May–2023 May	Sexual health clinic	Pharyngeal swabs, anal swabs	1,586 (93.9)	18	1,689
Grabmeier-Pfistershammer et al. 2024 (*4*)	Retrospective cohort	Austria	2023 Jan–2023 Mar	Sexual health clinic	Anal swabs	90 (100)	0	90
Hampel et al. 2023 (*20*)	Prospective cohort	Switzerland	2022 Aug–2022 Oct	SwissPrEPared program	Pharyngeal swabs, anal swabs	198 (98.5)	1	201
Koppe et al. 2024 (*21*)	Cross-sectional	Germany	2022 Apr–2023 Oct	Community center	Anal swabs, urethral swabs		75	6,600
Mizushima et al. 2023 (*22*)	Prospective cohort	Japan	2023 Jan–2023 Mar	Unknown	Anal swabs, urine	1,346 (100)	3	1,346
Ogale et al. 2023 (*23*)	Prospective cohort	USA	2022 Aug	Health clinic	Pharyngeal swabs, anal swabs, blood	359 (66.1)	2	543
Pestel et al. 2022 (*5*)	Prospective cohort	Germany	2022 Jun–2022 Jul	Infectious disease clinic	Pharyngeal swabs, anal swabs	53 (100)	0	53
Pitt-Kendall et al. 2023 (*9*)	Retrospective cohort	England	2022 Aug–2022 Oct	Sexual health clinic	Pharyngeal swabs, anal swabs, urine	1,159 (100)	2	1,159
Rossotti et al. 2023 (*6*)	Prospective cohort	Italy	2022 Sep–2022 Oct	Sexual health clinic	Pharyngeal and anal swabs	72 (100)	0	72
Thomassen et al. 2024 (*7*)	Prospective cohort	Denmark	2022 Sep–2022 Oct	Danish PrEP database	Anal swabs	224 (100)	0	224
Van Dijck et al. 2023 (*8*)	Mixed cohort	Belgium	2022 Jun–2022 Sep	Sexual health clinic	Anal swabs	327 (100)	0	327

*MPXV, monkeypox virus; PrEP, preexposure prophylaxis.

Cumulatively, of 14,511 persons who were tested for MPXV by PCR, 128 persons (weighted by the study’s inverse variance) had asymptomatic MPXV. Results of the Egger’s test to evaluate for publication bias was not statistically significant (p = 0.08), also yielding a symmetric funnel plot. Combined, those findings offered no significant evidence of publication bias having occurred and provided sufficient evidence to proceed with a random-effects analysis (Appendix Figure) (*12*). The ι^2^ value was 82.6% (95% CI 72.9%–88.8%), indicating a high level of heterogeneity between studies. The random-effects analysis yielded a final pooled prevalence of 1.0% (95% PI 0.1%–11.2%) (Figure 2).

**Figure 2:**
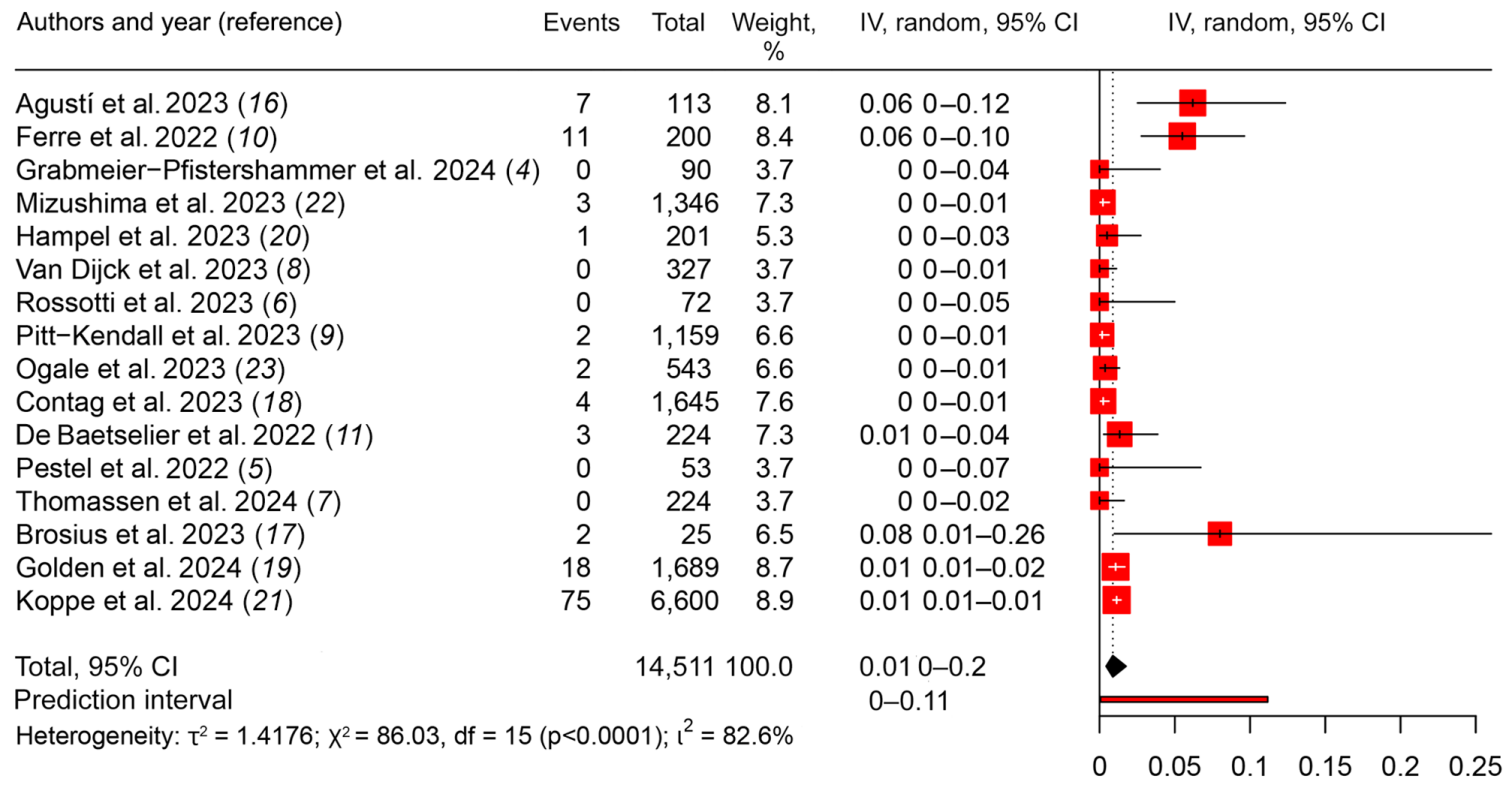
Forest plot of 16 studies eligible for inclusion to assess the pooled prevalence in a meta-analysis of asymptomatic monkeypox virus detections in nonendemic regions. Red blocks indicate point estimate; size of box indicates statistical weight of study. df, degrees of freedom; IV, inverse variance weighting.

We included all 16 studies in the meta-regression analysis of geographic region and study design. We observed no statistically significant difference in the odds of asymptomatic MPXV detections in Asia (odds ratio [OR] 0.45 [95% CI 0.02–10.03]) and Europe (OR 1.70 [95% CI 0.29–10.05]) compared with the odds in North America. In our assessment of the odds of asymptomatic MPXV detections by study design, we found no significant difference in the odds of MPXV detections in mixed cohorts (OR 0.06 [95% CI 0.00–3.94]), prospective cohorts (0.17 OR [95% CI 0.02– 1.20]), and retrospective cohorts (OR 0.24 [95% CI 0.03–1.83]), all relative to the odds of MPXV detections in cross-sectional studies (Table 2).

**Table 2:** Odds ratios and 95% CIs for meta-regression analysis of asymptomatic monkeypox virus detections in nonendemic regions, by geographic region and study design

Meta-regression variable	Odds ratio (95% CI)
Asia vs. North America	0.45 (0.02–10.03)
Europe vs. North America	1.70 (0.29–10.05)
Mixed cohort vs. cross-sectional	0.06 (0.00–3.94)
Prospective cohort vs. cross-sectional	0.17 (0.02–1.20)
Retrospective cohort vs. cross-sectional	0.24 (0.03–1.83)

## Discussion

We assessed the prevalence of asymptomatic MPXV detections during the global outbreak of clade IIb MXV in nonendemic regions that has been ongoing since 2022. We included 16 peer-reviewed studies that encompassed 14,511 adults of both sexes (95.0% of all participants were men), sexual orientations, races, and ethnicities who were tested for MPXV. The pooled prevalence of asymptomatic MPXV detections in this population was 1%, much lower than the previously reported pooled prevalence of 10% reported in a previous meta-analysis that included a large proportion of cases from Cameroon (where MPXV is endemic). We found that asymptomatic MPXV prevalence did not differ by study design or geographic region.

Most of the 16 studies we included were conducted in countries in Europe and were either prospective or retrospective analyses. The prevalence of asymptomatic MPXV detections in all 16 studies ranged from 0% to 8%; mean prevalence was 1.55%. Studies with larger sample sizes were weighted more heavily in our analysis, which led to a lower pooled prevalence of asymptomatic MPXV detections. The prospective study conducted by Brosius et al. (*17*) found the highest prevalence of asymptomatic MPXV among tested persons, probably because they restricted their sample to persons reporting sexual or intimate contact with someone with MPXV, whereas all other studies sampled from sexually active populations (largely those attending sexual health clinics); some, but not all, reported contact with a person with MPXV.

The low prevalence of asymptomatic MPXV detections observed in our study may be, in part, attributable to the increasing number of persons who have been previously infected with or vaccinated against MPXV with modified vaccinia Ankara–Bavarian Nordic vaccine, the latter of which may reduce the risk for infection from 75% to 86% (depending on the number of doses received) and reduces the severity of MPXV disease if the patient is infected (*24*,*25*). Because studies have shown that prophylactic vaccination with modified vaccinia Ankara–Bavarian Nordic vaccine reduces the severity of disease, vaccinated persons who become infected with MPXV may experience symptoms that are so mild that they may be classified as asymptomatic (and, especially in the United States, because of the absence of non–lesion-based testing, go undetected) (*26*,*27*). Persons who are asymptomatic or minimally symptomatic also may be less likely to seek medical attention, and the most ubiquitous assays to evaluate MPXV in clinical settings require an active lesion to swab for viral testing. However, the studies included in this analysis did not require lesions for testing; detection was assessed through PCR testing of oropharyngeal, rectal, or urethral swab samples or of urine or blood, and most of those studies evaluated high-risk groups (e.g., men at sexual health clinics).

One major strength of this meta-analysis was the inclusion of 16 different studies. We found a low pooled prevalence of asymptomatic MPXV detections among the entire pooled study population; however, that finding may be an underestimate because of underreporting of asymptomatic MPXV. Although the findings are reassuring, because we did not evaluate infectivity or transmission potential among asymptomatic persons, more accurate and widely available diagnostic MPXV testing tools (e.g., oropharyngeal swabs, as recommended by the World Health Organization) would help to better characterize the epidemiology and virology of MPXV from a surveillance standpoint, even if the use of those testing tools would be unlikely to change clinical practice (*1*,*26**,**28*).

Another strength of our meta-analysis is the inclusion of studies only relevant to the ongoing MPXV outbreak (i.e., those conducted since the outbreak began in May 2022). Because our analysis excluded studies conducted before the most recent MPXV outbreak began, we can be sure that our findings are a closer representation of the actual proportion of current asymptomatic MPXV infections than the findings from the prior meta-analysis that included studies conducted before 2022 (*2*). Finally, because antibody-based testing cannot be used to distinguish between past and current MPXV infection (or prior vaccination against MPVX), our meta-analysis includes only studies that performed PCR tests because the World Health Organization declared PCR tests the standard for diagnosing MPXV infection in humans (*26*).

One limitation of our analysis is that some persons might not have been followed long enough to have onset of symptoms or to be infected with MPXV; Rossotti et al. (*6*) only followed their study population for 21 days. Patients may have remained presymptomatic throughout the 21-day study period, experiencing symptom onset later. Because studies included in this analysis did not differentiate between presymptomatic and persistently asymptomatic infections, some persons may have been characterized as presymptomatic rather than persistently asymptomatic. In addition, we defined asymptomatic patients as persons without any physical symptoms (*29*). However, no formal definition of asymptomatic MPXV exists; asymptomatic MPXV infection in some instances could refer to a lack of lesions or rashes, a lack of classical MPXV infection symptoms, or a lack of physical symptoms entirely. A concrete definition of asymptomatic MPXV infection is needed to ensure a complete understanding. This limitation is not unique to MPXV infection and has been described among other viral infections (*30*,*31*).

Because only 5 studies in this meta-analysis included vaccination status of participants, we could not differentiate whether patients were asymptomatic or if they experienced milder nonlesion symptoms (e.g., febrile illness) because of attenuation of illness by vaccination. Future epidemiologic studies ideally will involve serologic MPXV antibody testing to more accurately assess the prevalence of previous (possibly asymptomatic) MPXV infection while also controlling for prior vaccination status. This approach is necessary because a limited number of serologic assays can differentiate antibodies specifically derived from MPXV infection from antibodies derived from other orthopox infections. In addition, because PCR testing can detect nonviable genetic material, future studies will need to assess the prevalence of replication-competent virus among asymptomatic persons who test positive for MPXV (*32*).

Most of the studies included in this analysis sampled highly selected populations, including GBMSM, sexual health clinic or vaccine clinic attendees, and contacts of persons with known MPXV infection. Such selection bias limits our interpretation of results to those populations, rather than the population of all adults in nonendemic countries.

The high level of heterogeneity among studies, indicated by the ι^2^ value and large 95% CI (82.6% [95% CI 72.9%–88.8%]), indicates important variability, probably attributable to differences in study populations, recruitment settings, definitions of asymptomatic infection, specimen collection methods, and laboratory testing approaches. The high level of heterogeneity also may have distorted our results from the Egger’s test of publication bias and could have resulted in an underpowered test. Given this variability and potential for an underpowered test, the pooled estimate should be interpreted with caution.

Finally, understanding the prevalence of asymptomatic MPXV detections does not help to elucidate the role of asymptomatically infected persons in MPXV transmission dynamics; in fact, MPXV detection in the absence of symptoms may not necessarily translate to infection (i.e., someone may have been recently exposed and have residual virus present but may not actually become infected). Whether persons who are asymptomatic (i.e., no symptoms whatsoever) can transmit infection, or whether persons who do not have lesions but do have respiratory or other prodromal symptoms are able to transmit infection, remains unclear. Previous studies already have demonstrated that transmission is possible when mild or atypical symptoms are present and that presymptomatic or asymptomatic transmission plays a vital role in other viral disease dynamics (*30*,*33*–*36*). We could not ascertain the prevalence of asymptomatic MPXV infections effectively in 2024 and 2025 because most of MPXV infections during the global outbreak outside of sub-Saharan Africa occurred in summer and fall of 2022. In California, 88% of MPXV cases during May 12, 2022–September 13, 2024, occurred in 2022.

Of note, we only summarize clade IIb MPXV infections in this article. Recent travel-imported cases of clade Ib MPXV underscore the complexity of MPXV clinical manifestations in relation to traditional symptoms and screening (*37*). Although clade Ib MPXV can be more pathogenic and infectious than clade IIb, whether clade Ib MPXV has the same low asymptomatic MPXV prevalence as clade IIb remains to be seen (*38*).

We found a low pooled study prevalence of asymptomatic clade IIb MPXV detections during May 12, 2022–September 13, 2024, outside of sub-Saharan Africa. Although the findings are reassuring, we did not evaluate infectivity or transmission potential among asymptomatic persons. MPXV continues to circulate in the United States overall and in California; the viral dynamics that enable its persistence remain poorly understood and merit further study. In unvaccinated persons, especially those living with HIV, mpox can cause serious disease and death, underscoring the importance of ongoing surveillance, vaccination, and vigilance for this disease.

Appendix Additional information about meta-analysis of asymptomatic monkeypox virus prevalence in nonendemic regions, 2022–2024.
