# Supplementary material for: Meta-analysis of Asymptomatic Monkeypox Virus Prevalence in Nonendemic Regions, 2022–2024
Source: Emerg Infect Dis. 2026 Oct;32(10):1590–8. doi: 10.3201/eid3210.260305 (PMC13632830; doi:10.3201/eid3210.260305)
Supplement: Supplementary file 1 [file 26-0305-Techapp-s1.pdf]

Article DOI: https://doi.org/10.3201/eid3210.260305

EID cannot ensure accessibility for supplementary materials supplied by authors. Readers who have difficulty accessing supplementary content should contact the authors for assistance.

Meta-analysis of Asymptomatic Monkeypox Virus Prevalence in Nonendemic Regions, 2022–2024

Appendix

Appendix Table. Joanna Briggs Institute critical appraisal checklist for prevalence studies

Study	Sample frame	Sample participants	Sample size	Subjects and setting	Data analysis	Valid methods	Standard measurement	Statistical analysis	Response rate
Agusti et al. 2023	Yes	Yes	Yes	Yes	Yes	Yes	Yes	Yes	No
Ferre et al. 2022	Yes	Yes	Yes	Yes	Yes	Yes	Yes	Yes	Unclear
Grabmeier-Pfistershammer et al. 2023	Yes	Yes	Yes	Yes	Yes	Yes	Yes	Yes	Unclear
Mizushima et al. 2023	Yes	Yes	Yes	Yes	Yes	Yes	Yes	Yes	Unclear
Hampel et al. 2023	Yes	Yes	Yes	Yes	Yes	Yes	Yes	Yes	Unclear
Van Dijck et al. 2022	Yes	Yes	Yes	Yes	Yes	Yes	Yes	Yes	Unclear
Rossotti et al. 2023	Yes	Yes	Yes	Yes	Yes	Yes	Yes	Yes	Unclear
Pitt-Kendall et al. 2023	Yes	Yes	Yes	Yes	Yes	Yes	Yes	Yes	Unclear
Ogale et al. 2023	Yes	Yes	Yes	Yes	Yes	Yes	Yes	Yes	Unclear
Waddell et al. 2023	Yes	Yes	Yes	Yes	Yes	Yes	Yes	Yes	Unclear
Contag et al. 2023	Yes	Yes	Yes	Yes	Yes	Yes	Yes	Yes	Unclear
Baetselier et al. 2022	Yes	Yes	Yes	Yes	Yes	Yes	Yes	Yes	Unclear
Pestel et al. 2022	Yes	Yes	No	Yes	Yes	Yes	Yes	Yes	Unclear
Thomassen et al. 2024	Yes	Yes	Yes	Yes	Yes	Yes	Yes	Yes	Unclear
Minhaj et al. 2023	Yes	Yes	Yes	Yes	Yes	Yes	Yes	Yes	Unclear
Brossius et al. 2022	Yes	Yes	No	Yes	Yes	Yes	Yes	Yes	Unclear
Golden et al. 2024	Yes	Yes	Yes	Yes	Yes	Yes	Yes	Yes	Unclear
Pathela et al. 2024	Yes	Yes	Yes	Yes	Yes	Yes	Yes	Yes	Yes
Koppe et al. 2024	Yes	Yes	Yes	Yes	Yes	Yes	Yes	Yes	Unclear

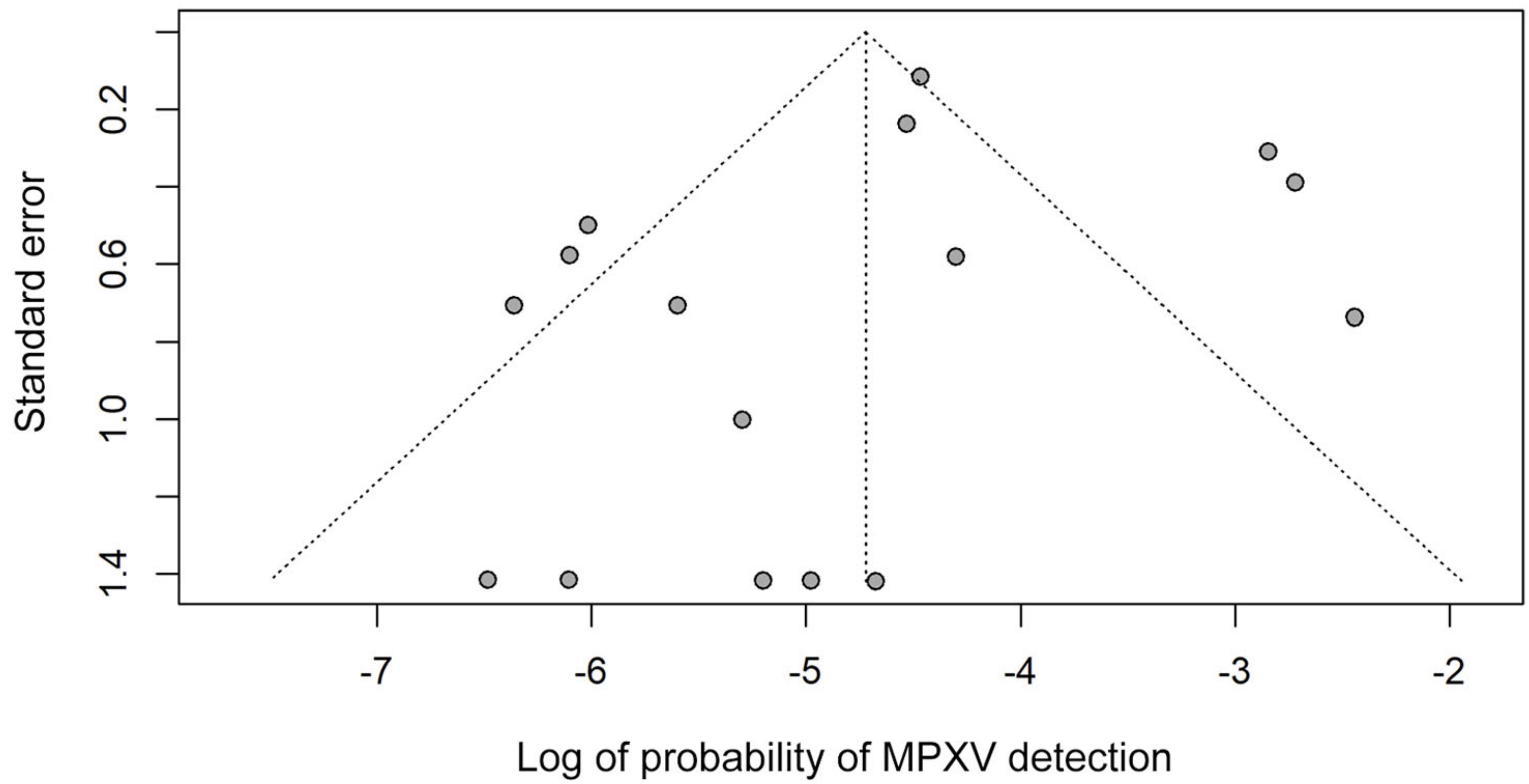

Appendix Figure. Funnel plot of research studies included in meta-analysis of asymptomatic mpox virus (MPXV) detections.
